# Editorial: Animal-computer interaction and beyond: The benefits of animal-centered research and design

**DOI:** 10.3389/fvets.2022.1109994

**Published:** 2023-01-04

**Authors:** Clara Mancini, Eleonora Nannoni

**Affiliations:** ^1^Animal-Computer Interaction Lab, School of Computing and Communications, The Open University, Milton Keynes, United Kingdom; ^2^Department of Veterinary Medical Sciences, University of Bologna, Bologna, Italy

**Keywords:** animal research, animal-centered research ethics, beyond the 3rs, research ethics principles, ethical scoring system

Animals are increasingly exposed to interactive technologies and involved in technological interactions. Ambient and wearable devices are now frequently used by humans to monitor animals' states and behaviors within conservation or husbandry practices, and a growing number of animals engage with interactive or technological systems as a part of their activities in research laboratories, on farms, in zoos and in domestic environments.

The field of Animal-Computer Interaction (ACI) investigates how interactive technologies affect the individual animals involved; what technologies could be developed, and how they should be designed in order to improve animals' welfare, support their activities and foster positive interspecies relationships; and how research methods could enable animal stakeholders to participate in the development of relevant technologies. A fundamental tenet of ACI is that the technologies being designed, the methods employed for their development and the ethical underpinnings of those methods should be animal-centered, meaning that the characteristics, needs and wants of the animals involved should be of primary consideration and should directly inform design processes and outcomes. This perspective has key advantages, for example, ensuring that animals' interactions with technologies are positive and effective, and addressing growing societal concerns over the treatment of animals.

This Research Topic collects contributions from different perspectives, which highlight possible animal-centered approaches in research and design, as well as challenges that animal-centered approaches might pose and how these might be addressed. Bringing together novel contributions that demonstrate how animal-centered technologies, research methods and ethical frameworks could benefit research and practice in different domains - including farming, animal conservation and welfare, or animal research - this e-collection provides a resource for researchers and practitioners whose work involves animals and for whom the applicability of animal-centered technologies, methods or frameworks may be relevant.

Frameworks that aim to support animal-centered design processes have the potential to enable animal agency and enhance their welfare, as discussed by Webber et al.. In their proposed framework, the authors combine the “Five Domains of Animal Welfare” model and the “Coe Individual Competence” model, providing a structured approach to defining and refining animal-centric objectives to design technologies that can promote positive animal welfare in managed settings. Throughout the process, animal-centered design involves paying close attention to the sensory, cognitive and physical characteristics of the animals in question. In this regard, drawing together academic perspectives from ecology, neuroscience, anthropology, philosophy, interaction design, and arts, French argues for expanding the aesthetic dimensions of design beyond the limits of human capability to encompass other species' sensory modalities and include non-human aesthetic sensibilities. Likewise, Carter et al.'s work highlights the importance of considering animals' ergonomics when designing artifacts that they are expected to interact with. Examining canine working trials, the authors measure vertical forces and apparent joint angulation at landing in dogs traversing a scale of different heights, suggesting that the maximum scale height should be reviewed to minimize impacts on the physical health and welfare of participating dogs. However, the impacts that technological interventions may have on animals are not limited to physical interactions and, in this regard, Paci et al. discuss the importance that privacy has for animals. The authors draw from observations of privacy-related behaviors in different species, finding that animals use a variety of distance regulation and information management mechanisms to secure their own and their assets' safety, and to negotiate social interactions. Thus, they argue that the design of interactive systems needs to be informed by animals' privacy requirements.

Given the interspecies communication barriers and power asymmetries characterizing human-animal relations, understanding, let alone prioritizing, animals' requirements poses significant, emergent and often unexpected ethical challenges. To help researchers deal with such challenges, Ruge and Mancini propose an ethics toolkit for clearly and systematically articulating the ethical stance both of researchers and of the projects researchers work on, to support moment-by-moment decision-making. An implication of animal-centered research and design is that decisions related to processes and outcomes should prioritize animals' interests, with regards to both research outcomes and processes. Exploring the applicability of such a perspective to all animal research, Mancini and Nannoni propose an ethical framework for conducting research *with* animals, highlighting the principles of relevance, impartiality, welfare and consent, and provide a scoring system to help researchers and delegated authorities assess research procedures, with a view to shifting research practices toward more animal-centered approaches.

Alongside the abovementioned proposals, contributions based on novel technological applications demonstrate the potential benefits of animal-centered research and design, for example, to analyse animals' behavior and achieve a more objective understanding of their abilities and needs. Using sensor-instrumented dog toys to test dogs undergoing advanced training to become service dogs, Bryne et al. discovered that a measure of average bite duration could help predict a dog's success as a service dog. Therefore, they suggest the use of instrumented toys in addition to current behavioral assessments. Similarly, Menaker et al. demonstrate how, consistent with questionnaire-based assessments, the application of unsupervised machine learning techniques could help cluster dogs' responses during standard behavioral tests and, thus, support the early exploration of behavioral data before forming and testing behavioral research hypotheses. Moreover, machine learning can be applied to understand animals' interactions with environments shared by multiple individuals. To this end, the facial recognition system developed by Brookes et al. measured how individual members in a troop of seven zoo-housed gorillas used cognitive enrichment equipment, effectively recognizing individual gorillas. To support the automatic, real-time evaluation of cognitive enrichment interventions, the authors propose the integration of sensors that could record the animals' detailed interaction with specific elements of the equipment.

Machine vision combined with Internet of Things (IoT) systems have significant potential also for managing and optimizing animal farming conditions. To support automatic processing within Black Soldier Fly and the domestic cricket farming, Hansen et al. used object detection and classification techniques to count and size fly larvae and to sex crickets, as well as IoT technology to monitor various environmental parameters and thus maintain suitable conditions. Furthermore, Neethirajan suggests how novel uses of technologies such as deepfake could help improve the welfare of farmed animals if used to generate large video datasets on which to train machine learning models that can accurately monitor animal health and identify their emotional state; and maybe even enable interventions that could ameliorate animal behavior, for example by displaying digital conspecifics. Completing this series of contributions, Bendel looks into future developments of partially and fully autonomous machines and robots, discussing how they could be designed to avoid harming or to protect animals. The author divides these systems into passive (e.g., systems that detect the presence of animals), active (e.g., systems that feed animals) and proactive (e.g., fully automated systems that protect animals); he discusses how these could be designed, providing numerous examples for each category.

Overall, the 12 contributions in this Research Topic provide a rich overview of cutting-edge animal-centered design approaches and applications of animal-centered technologies within domains as diverse as animal farming, research, conservation, and welfare. Readers will find discussions on the benefits that the proposed approaches and technologies have or could have for the welfare of animals, for the activities in which they are involved, and for human-animal relations, as well as discussions on challenges to the applicability of animal-centered perspectives and how such challenges might be addressed.

## Author contributions

Both authors listed have made a substantial, direct, and intellectual contribution to the work and approved it for publication.

